# The BCL2/BAX/ROS pathway is involved in the inhibitory effect of astragaloside IV on pyroptosis in human umbilical vein endothelial cells

**DOI:** 10.1080/13880209.2022.2101668

**Published:** 2022-09-19

**Authors:** Yi Su, Xin Yin, Xin Huang, Qianqian Guo, Mingyuan Ma, Liheng Guo

**Affiliations:** a Department of Critical Care Medicine, Foshan Hospital of Traditional Chinese Medicine, Guangzhou University of Chinese Medicine, Foshan, China; b The Second Clinical College of Guangzhou University of Chinese Medicine, Guangzhou, China; c Department of Critical Care Medicine, The Second Affiliated Hospital of Guangzhou University of Chinese Medicine, Guangzhou, China

**Keywords:** Sepsis, lipopolysaccharide, mitochondria, inflammasome, endothelial injury

## Abstract

**Context:**

Astragaloside IV (AS-IV) is extracted from *Astragalus membranaceus* (Fisch.) Bunge (Fabaceae). However, its effects on endothelial cell injury remain unclear.

**Objective:**

To investigate the mechanisms underlying the effects of AS-IV on lipopolysaccharide (LPS)-induced endothelial injury *in vitro*.

**Materials and methods:**

Human umbilical vein endothelial cells (HUVECs) were pre-treated with AS-IV (100 µmol/mL), 4-hydroxy-3-methoxyacetophenone (APO, 10 µmol/mL), *N*-acetylcysteine (NAC, 50 µmol/mL) and Ac-YVAD-cmk (AC, 5 µmol/mL) for 2 h before 1 μg/mL LPS 24 h exposure. Untreated cells cultured without any exposure were used as controls. Cell viability, reactive oxygen species (ROS) and pyroptosis assays were performed. The pyroptosis related proteins were detected by western blot.

**Results:**

The rate in late pyroptosis (Q2-2) of AS-IV (13.65 ± 0.74%), APO (13.69 ± 0.67%) and NAC (15.87 ± 0.46%) groups was lower than the LPS group (21.89 ± 0.66%, *p* < 0.05), while the rate in early pyroptosis (Q2-4) of AS-IV group (12.00 ± 0.26%) was lower than other groups (*p* < 0.05). The expression of NOX4, GSDMD, NLRP3, ASC and caspase-1 decreased after AS-IV, NAC or AC intervention (*p* < 0.05). The ROS production in AS-IV (4664 ± 153.20), APO (4094 ± 78.37), NAC (5103 ± 131.10) and AC (3994 ± 102.50) groups was lower than the LPS (5986 ± 127.30) group, while the mitochondrial BCL2/BAX protein expression ratio increased in AS-IV, APO and NAC groups (*p* < 0.05).

**Discussion and conclusions:**

AS-IV suppressed pyroptosis in LPS-activated HUVECs by inducing ROS/NLRP3-mediated inhibition of the inflammatory response, providing a scientific basis for clinical applications of AS-IV.

## Introduction

Sepsis is a disorder of the host response to infections and leads to life-threatening organ dysfunction; it affects millions of people each year and is one of the major causes of death worldwide (Coopersmith et al. 2018). The pathophysiological features of septic shock are vasodilation, increased vascular permeability, decreased blood volume and ventricular dysfunction. The normal endothelium can limit the bacterial spread and orchestrate leukocyte recruitment and bacterium elimination (Joffre et al. 2020). However, endothelial phenotypic changes and endothelial dysfunction are important factors in sepsis, often leading to hypotension, insufficient organ perfusion, shock and death, partly due to acute vascular dysfunction and leakage (Russell et al. 2018). In addition, endothelial barrier dysfunction and microvascular leakage are important causes of organ failure in sepsis and sepsis-related complications such as acute lung injury (Opal and van der Poll 2015). Endothelial injury is a common pathophysiological feature of septic shock.

The exact mechanisms of cell damage and organ dysfunction caused by sepsis are not yet fully understood. Tissue hypoxia, mitochondrial dysfunction and apoptosis are considered critical mediators of sepsis shock. Organ dysfunction is an important predictor of patient prognosis, and multiple organ dysfunction is associated with a higher risk of death (Cecconi et al. 2018). Recent studies reported that mitochondria could release mitochondrial reactive oxidative species (mtROS), leading to the activation of the NLRP3 inflammasome (Alfonso-Loeches et al. 2014; Rovira-Llopis et al. 2018; Mohanty et al. 2019; Xu et al. 2019).

Indeed, the nucleotide-binding domain leucine-rich repeat-containing receptor (NLR) family protein NLRP3 plays a key role in host defence. It can be activated by many pathogen-derived, environmental and host-derived factors, including bacteria, viruses, fungi, dying cell components and crystal particles. Accumulating evidence suggests that the inflammasomes are involved in the pathogenesis of sepsis, especially NLRP3 (Wu et al. 2021). Inflammasomes trigger pyroptosis in a caspase-1-dependent manner (Xue et al. 2019). Pyroptosis is a rapid cell lysis mechanism following infection (Zychlinsky et al. 1992; Cookson and Brennan 2001) and involves endothelial cells and vascular smooth muscle cells (Pan et al. 2018; Zhang et al. 2018). Knockout of caspase-1 or caspase-11 protects mice from endotoxic shock (Li et al. 1995; Hagar et al. 2013; Kayagaki et al. 2015). The cytoplasmic delivery of lipopolysaccharide (LPS) activates caspase signalling and pyroptosis (Man et al. 2016; Vanaja et al. 2016). RIPK3 is also involved in pyroptosis (Grootjans et al. 2017). Caspase-11 activates gasdermin D, leading to pores in the cell membrane and lytic death by cell swelling (Broz 2015; Jorgensen and Miao 2015). Pyroptosis is involved in endothelial dysfunction and injury during sepsis (Singla and Machado 2018; Peng et al. 2020).

Astragaloside IV (AS-IV) is a compound extracted from *Astragalus membranaceus* (Fisch.) Bunge (Fabaceae) which is widely used in traditional Chinese medicine. AS-IV possesses protective effects on the heart, vasculature, lung, kidney and brain due to its potent antioxidant, anti-inflammatory, anti-apoptotic and immune-boosting effects (Zhang et al. 2020). In addition, AS-IV can modulate multiple pathways involved in cell life and death (Zhang et al. 2020). Moreover, it can attenuate sepsis-induced intestinal barrier dysfunction by preventing oxidative stress inflammation and activating the NLRP3 inflammasome (Zhang and Frei 2015; Li et al. 2016; Zhu et al. 2019; Xie et al. 2020).

Human umbilical vein endothelial cells (HUVECs) can be used to establish experimental models of vascular inflammation *in vitro*, and LPS-induced injury in HUVECs is commonly used as an *in vitro* model of sepsis (Hou et al. 2019). Due to the key role of NLRP3 inflammasome and mitochondria in septic shock, this study exams the role of AS-IV in regulating mitochondrial function and inhibiting NLRP3 inflammation activation. The results could help further understand the pathophysiology of sepsis and identify new potential treatment targets.

## Materials and methods

### Cell culture and drug treatment

HUVECs were purchased from the Type Culture Collection of the Chinese Academy of Sciences (Shanghai, China). HUVECs were cultured in DMEM medium containing 1% penicillin/streptomycin and 10% foetal bovine serum (FBS) (GIBCO, Invitrogen Inc., Carlsbad, CA) at 37 °C in a 5% CO_2_ incubator. The HUVECs were passaged when fused into a monolayer, using 0.125% pancreatin–0.02% EDTA digestion. The 3rd–5th generations of HUVECs were used for the experiments. LPS was used to induce HUVEC pyroptosis *in vitro*. The HUVECs were pre-treated with different drugs for 2 h before 1 μg/mL LPS 24 h exposure. AS-IV (purity: 98%) was provided by Shanghai TAUTOG Biotech Co., Ltd. (Shanghai, China). LPS, 4-hydroxy-3-methoxy acetophenone (APO) and *N*-acetylcysteine (NAC) were from Sigma-Aldrich (St. Louis, MO). Ac-YVAN-cmk (AC) was purchased from InvivoGen (Hong Kong, China). Cells cultured without any exposure and treatment were used as controls. The groups were (1) control, (2) LPS, (3) LPS + AS-IV (100 μmol/mL), (4) LPS + APO (10 μmol/mL), (5) LPS + NAC (50 μmol/mL) and (6) LPS + AC (5 μmol/mL).

### Cell viability assay

Cell viability was tested using the Cell Counting Kit-8 (CCK-8) (Dojindo, Kumamoto, Japan), as described by the manufacturer. First, HUVECs were cultured to reach the desired confluence in 96-well plates. Then, they were incubated with different treatments for 2 h at 37 °C, and CCK-8 was added to each well. Following incubation, a microplate reader (Tecan, Mannedorf, Switzerland) was used to detect the absorbance at 488 nm.

### Detection of ROS

The reactive oxygen species (ROS) levels were measured using the DCFH-DA Kit (BestBio, Shanghai, China), according to the manufacturer’s instructions. The cells (1 × 10^7^/mL) were labelled with 10 μM of the fluorescent probe DCFH-DA, followed by incubation for 20 min at 37 °C. Then, a serum-free cell culture medium was used for washing the cells three times. The fluorescence activity was analysed by flow cytometry. The amount of intracellular ROS was proportional to the fluorescence intensity.

### Detection of pyroptosis

Flow cytometry was used to detect pyroptosis using the Annexin V-FITC/PI apoptosis kit (Multi Sciences (Lianke) Biotech, Co., Ltd., Hangzhou, China), according to the manufacturer’s instructions. First, the cells were harvested and incubated at 37 °C in a 5% CO_2_ incubator after treatment. The unbound FLICA reagent was washed using PBS at the end of incubation. Next, the HUVECs were stained with 5 μL of Annexin V-FITC and 10 μL of PI for 5 min at 37 °C in the dark. Then, the HUVECs were analysed using flow cytometry (Agilent NovoCyte Quanteon, Santa Clara, CA). Pyroptotic cells were defined as double-positive for Annexin V-FITC and PI.

### Mitochondrial protein extraction

Mitochondrial proteins were extracted with the Cell Mitochondria Isolation Kit (Beyotime Biotechnology Co., Ltd., Shanghai, China), following the manufacturer’s instructions. The HUVECs were cultured in 75 mm culture vessels with 2 × 10^7^ cells in each vessel; then, they were incubated with different treatments for 24 h at 37 °C. Following incubation, the cells were collected using a trypsin–EDTA solution and centrifuged at 200 rpm at room temperature for 5 min. The cells were washed with pre-cooled PBS and centrifuged at 600 rpm at 4 °C for 5 min. The mitochondrial separation reagent containing phenylmethylsulfonyl fluoride (PMSF) was used to resuspend the cells, setting them on ice for 15 min. The cell walls were disrupted by ultrasound at 20 W, 10 s/time, until the trypan blue staining of the cells was ≥50%. The homogenized cells were centrifuged at 600 rpm at 4 °C for 10 min. The supernatant was centrifuged at 11,000 rpm at 4 °C for 10 min again. Mitochondrial lysate containing PMSF was resuspended.

### Western blotting

Cell lysates were prepared from HUVECs using a RIPA buffer that contained phosphatase inhibitors (Cayman Chemical, Ann Arbor, MI) and Halt protease inhibitors (Pierce, Rockford, IL). The BCA assay (Thermo Fisher Scientific, Waltham, MA) was used to determine the protein concentrations. The cell lysates were mixed with loading buffer and separated on 10% and 12% SDS polyacrylamide gels. The proteins on the gels were transferred to PVDF membranes. The membranes were blocked in TBST containing 5% BSA for 1 h. After blocking, the membranes were incubated overnight at 4 °C with the primary antibodies: anti-GAPDH (#5174S), anti-caspase-1 (#3866T), anti-GSDMD (#97558S), anti-BCL2 (#15071T), anti-BAX (#5023S) (all 1:1000, Cell Signaling Technology, Inc., Danvers, MA), anti-ASC (#sc-514414, Santa Cruz Biotechnology, Santa Cruz, CA), anti-NOX4 (#14347-1-AP, Proteintech Group Inc., Chicago, IL) and anti-NLRP3 (#ab263899, Abcam, Cambridge, UK). The membranes were washed in TBST and then incubated at room temperature for 2 h with goat anti-rabbit IgG (1:4000, Cell Signaling Technology, Inc., Danvers, MA). The membranes were rinsed three times with TBST, scanned, and analysed by ImageJ software (National Institutes of Health, Bethesda, MD).

### Statistical analysis

All statistical analyses were performed using GraphPad Prism 8 (GraphPad Software Inc., San Diego, CA). All data were presented as mean ± SEM. Student’s *t*-test was used to compare means between two groups, while ANOVA was used to compare multiple groups with Bonferroni’s *post hoc* test. The significance value was set at *p* < 0.05.

## Results

### Determination of the optimum drug concentrations

**Figure 1.**
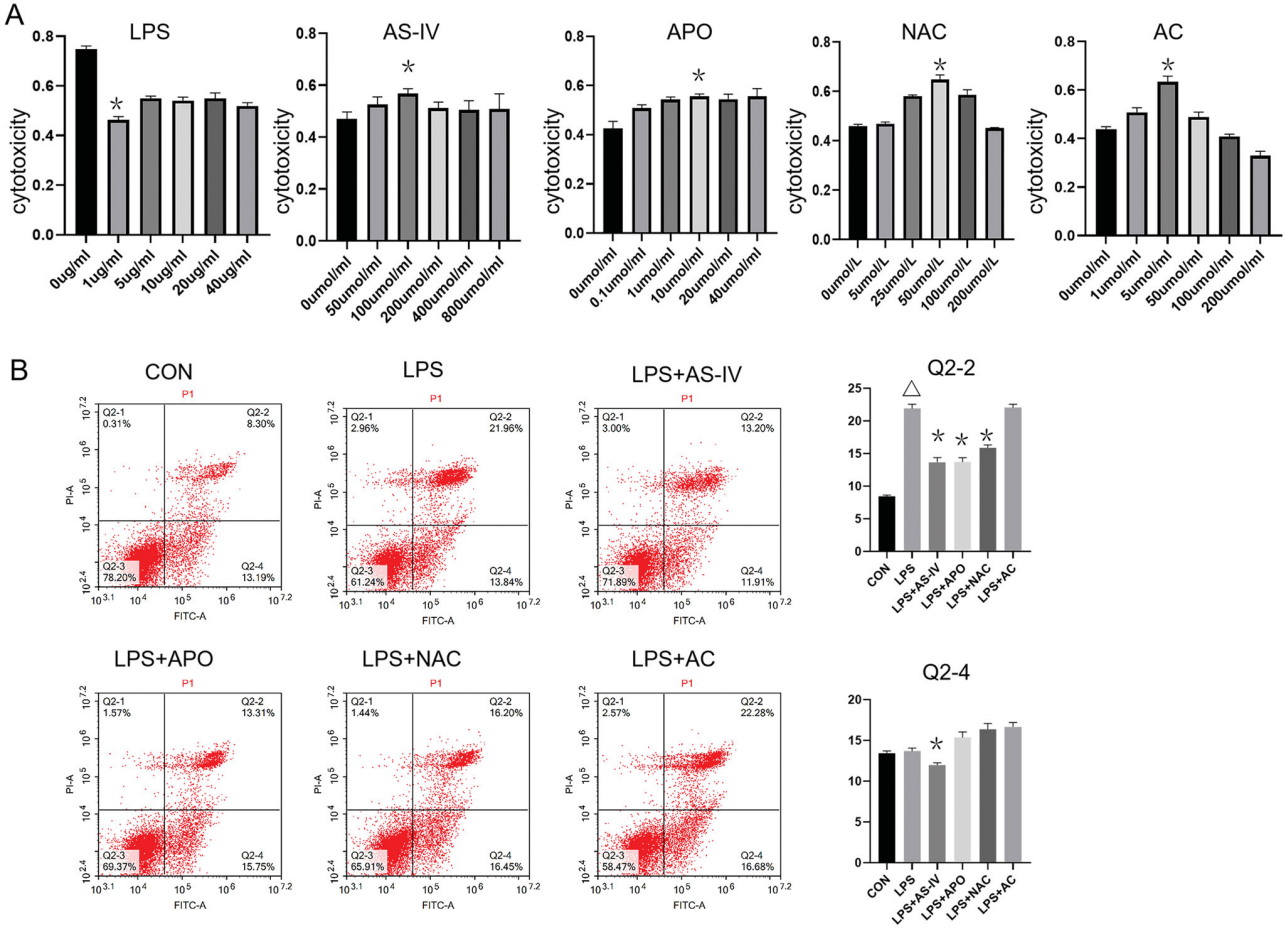
(A) Cytotoxicity and proliferation assay of drugs. LPS could inhibit the growth of HUVECs; 1 μg/mL was the appropriate concentration for inhibiting the growth of HUVECs for 24 h. Specific concentrations of AS-IV, APO, NAC and AC could help to increase the growth of HUVECs; 100 μmol/mL AS-IV, 10 μmol/mL APO, 50 μmol/mL NAC and 5 μmol/mL AC were the most appropriate concentration in increasing proliferation of HUVECs. *p < 0.05 vs. 0 group (*n* = 5). (B) Flow cytometry indicated that the median percentage of cells in late pyroptosis (Q2-2) was higher in the LPS group and lower in the AS-IV, APO and NAC groups. The median percentage of cells in early pyroptosis (Q2-4) in the AS-IV group was lower than in the other groups. *p < 0.05 vs. LPS group, ^Δ^*p* < 0.05 vs. control group (*n* = 4).

Different LPS, AS-IV, APO, NAC and AC concentrations were used on HUVECs in 96-well plates for 24 h. Using the CCK-8 assay, cytotoxicity and proliferation could be determined. LPS 1 µg/mL displayed the highest cytotoxic effect among the five concentrations tested. AS-IV 100 μmol/mL, APO 10 μmol/mL, NAC 50 μmol/mL and AC 5 μmol/mL was the most appropriate concentration for increasing HUVEC proliferation (Figure 1(A)). The results demonstrated the optimal drug concentrations for the subsequent experiments.

### AS-IV inhibited cell death

Cell death was detected by flow cytometry. LPS could induce late cell death. APO and NAC could reduce the late death of HUVECs. Furthermore, AS-IV could decrease both early and late death of HUVECs (Figure 1(B)). Hence, AS-IV can inhibit the death of HUVECs.

### AS-IV regulated pyroptosis via the NLRP3 inflammasome

**Figure 2.**
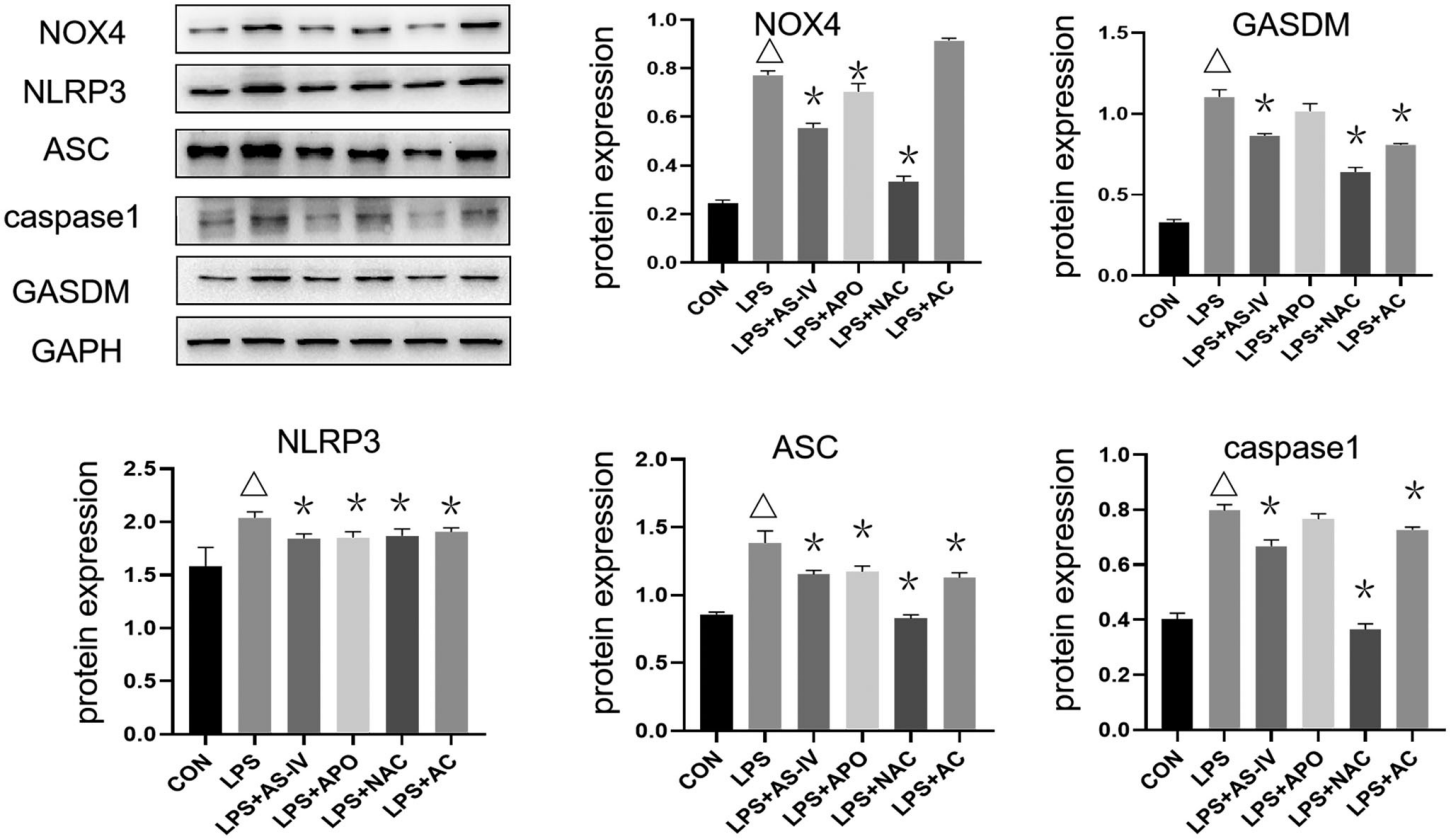
AS-IV regulated pyroptosis via the NLRP3 inflammasome. The protein expression of NOX4, GSDMD, NLRP3, ASC and caspase-1 was measured by western blot and was higher in the LPS group than in the CON group. The expression of NOX4, GSDMD, NLRP3, ASC and caspase-1 was lower in the AS-IV, NAC and AC groups than in the LPS group. *p < 0.05 vs. LPS group, ^Δ^*p* < 0.05 vs. control group (*n* = 4).

The protein expression levels of NOX4, GSDMD, NLRP3, ASC and caspase-1 were higher in the LPS group compared with the control group. On the other hand, the expression levels of NOX4, GSDMD, NLRP3, ASC and caspase-1 were lower in the AS-IV, NAC and AC groups than in the LPS group (all *p* < 0.05). The differences in NOX4 expression in the AC group and the expression of GSDMD and caspase-1 in the APO group were not significantly different from the LPS group (Figure 2). These results suggest that AS-IV can modulate key proteins involved in the NRLP3 inflammasome.

### AS-IV inhibited LPS from inducing ROS via the BCL2/BAX pathway

**Figure 3.**
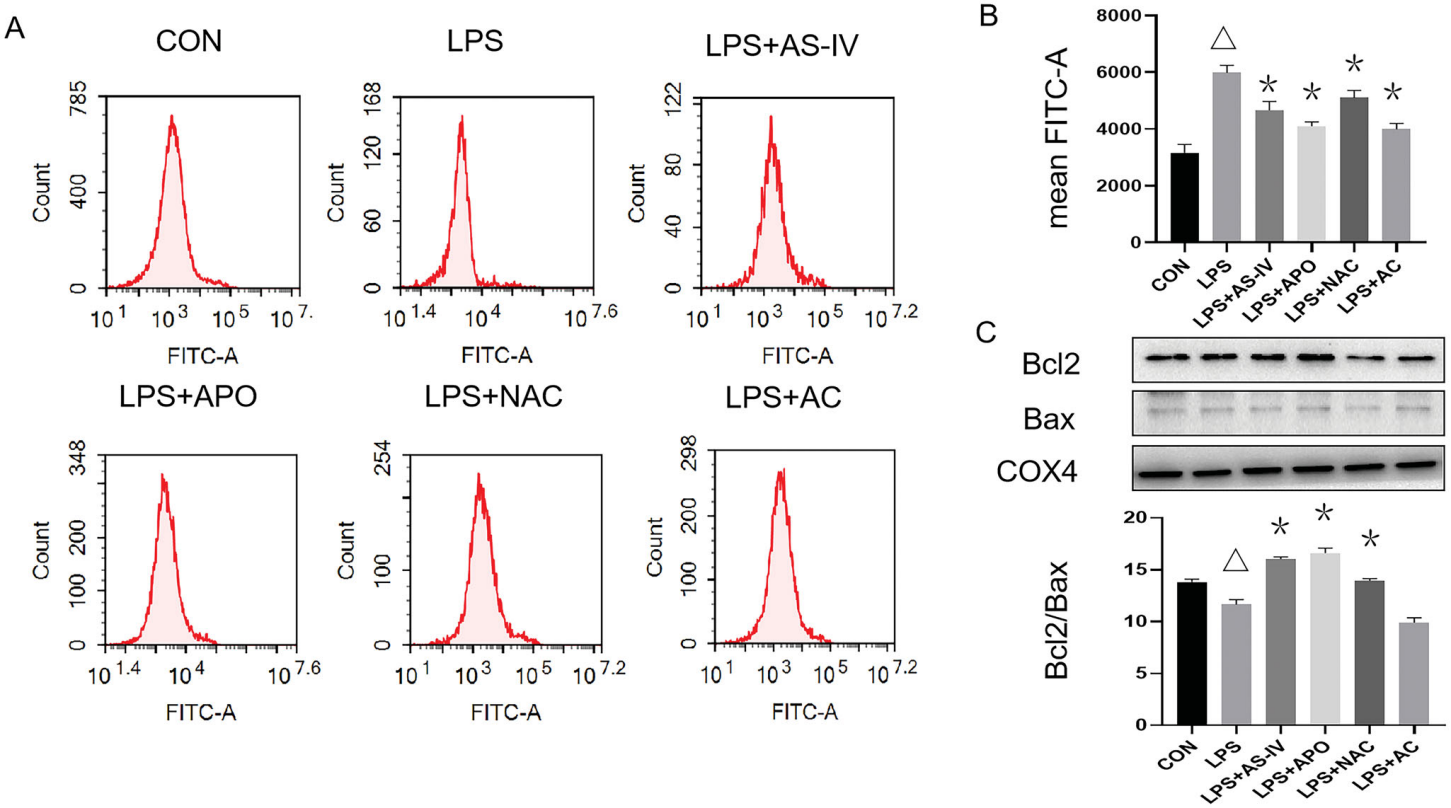
AS-IV inhibited LPS-induced ROS via BCL2/BAX pathway. (A, B) The production of ROS in the LPS group was higher than in the control group, while AS-IV, APO, NAC or AC groups’ ROS were lower than in the LPS group. (C) BCL2/BAX mitochondrial protein expression ratio was lower in the LPS group than in the CON group and higher in the AS-IV, APO and NAC group than in the LPS group. *p < 0.05 vs. LPS group, ^Δ^*p* < 0.05 vs. control group (*n* = 4).

LPS increased ROS production in HUVECs (Figure 3(A,B)). AS-IV, APO, NAC and AC decreased the production of ROS after LPS exposure (Figure 3(A,B)). The BCL2/BAX mitochondrial protein expression ratio was lower in the LPS group than that in the control group, while the ratio was higher in the AS-IV, APO and NAC groups than in the LPS group (all *p* < 0.05) (Figure 3(C)). Hence, it could be assumed that AS-IV might exert its protective effects by reducing ROS production.

## Discussion

Endothelial injury is a pathophysiological feature of septic shock. AS-IV has a protective effect against endothelial cell injury, but the specific mechanisms remain unclear. This study investigates the underlying mechanisms of AS-IV on LPS-induced endothelial injury *in vitro*. The results suggest that AS-IV might inhibit LPS-induced HUVECs pyroptosis via the mitochondrial BCL2/BAX and ROS/NLRP3 inflammasome pathways.

AS-IV possesses antioxidant, anti-inflammatory and anti-apoptotic effects that protect several organs, including the blood vessels (Zhang et al. 2020). AS-IV significantly enhances cell viability and migration in ox-LDL-induced HUVEC by inhibiting LDH release, apoptosis, ROS production and NADPH oxidase (NOX) (Zhu et al. 2019). In this study, AS-IV reduced ROS production. AS-IV significantly suppresses heat-induced apoptosis, as indicated by the increased expression of the pro-apoptotic genes BAK, BIK and BMF and increased expression of the apoptosis markers BAX, cleaved PARP, cleaved caspase-3 and cytochrome C (Dong et al. 2017). AS-IV inhibits the mRNA upregulation of Fas, FasL, caspase-8 and BAX/BCL2 (Yin et al. 2020), supporting the present study. AS-IV decreases the protein expression levels of NLRP3, caspase-1, ASC, IL-18, IL-1β and calpain-1 *in vivo* and/or *in vitro* in a pulmonary hypertension model (Sun et al. 2021).

Oxidative stress plays a major role in the progression of endothelial injury. The NOX family proteins are the main source of ROS in cardiovascular diseases and can cause the oxidative inactivation of NO, which leads to the decoupling of eNOS and continuous oxidative stress state. There are four subtypes of NOX expressed in endothelial cells (NOX1, NOX2, NOX4 and NOX5) (Jiang et al. 2020). Exogenous H_2_O_2_ or overexpression of NOX4 (which produces H_2_O_2_) increases mtROS (Kim et al. 2017). Excess mtROS weakens the mitochondrial membrane potential (MMP), which influences the opening of the mitochondrial permeability transition pore (mPTP), and the activation of the ROS release mechanism, inducing ROS burst and mitochondrial dysfunction and damaging the vascular endothelium (He et al. 2019).

Little information about the interaction between mitochondrial dysfunction and inflammasome in endothelial injury is available. Likewise, the cellular source of inflammasome activation during endothelial injury is lacking. This study revealed that HUVECs expressed LPS-induced NLRP3 inflammasomes. LPS triggered the recruitment of NLRP3 and activation of caspase-1 by inducing ROS formation. Furthermore, this study identified the interaction between NOX4, ROS, and the NLRP3 inflammasome complex, i.e., the inhibition of NOX4 and ROS might significantly reduce the NLRP3 inflammasome activation. The NLRP3 inflammasome is an intracellular protein complex composed of NLRP3, ASC and pro-caspase-1. Caspase-1 plays a key role in host defence through its dual function in inducing a pro-inflammatory cell death termed pyroptosis and promoting the secretion of pro-inflammatory cytokines (Brodsky and Medzhitov 2011) by cleaving and activating GSDMD (Orning et al. 2019). ROS production is also an important factor in activating the NLRP3 inflammasome (Alfonso-Loeches et al. 2014; Xu et al. 2019). Although the cellular and molecular mechanisms by which mtROS induces inflammasome activation remain to be determined, this study provided evidence that LPS induced HUVECs to express NLRP3 and promote ASC oligomerization, which allowed NLRP3 recruitment and caspase-1 activation. ROS production was decreased by AS-IV, APO and NAC, which downregulated NLRP3, ASC and caspase-1 expression, indicating that AS-IV might attenuate NLRP3 inflammasome activation and pyroptosis by inhibiting the production of ROS in HUVECs. This hypothesis is in agreement with the previous studies indicating that AS-IV can prevent NLRP3 activation (Zhang and Frei 2015; Li et al. 2016; Zhu et al. 2019; Xie et al. 2020).

Mitochondria are the major intracellular source of ROS. BCL2 is related to mtROS through its influence on the activity of mitochondrial complex IV to regulate, promote mitochondrial binding to GSH, and interact with small mitochondrial GTPase-Rac1 (Chong et al. 2014). This study demonstrated that LPS could increase BAX expression or decrease BCL2 expression. Besides, AS-IV and ROS inhibitors could reduce ROS production and increase the ratio of BCL2/BAX expression, which supported the role of the BCL2/BAX pathway in ROS production. The downregulation of the BCL2/BAX protein ratio and large ROS production might trigger the mitochondrial transition pore (MTP) to initiate the apoptosis process by releasing different molecules to convert apoptotic factors into the cytosol (Guo et al. 2013). Evidence suggested that LPS could aggravate H9C2 cell injury by triggering the mitochondrial stress response and ROS production, inducing NLRP3 inflammasome-mediated pyroptosis (Qiu et al. 2019). The cell innate immune response causes cell death by inflammasome activation. Proteolytic cleavage of GSDMD by caspase-1, caspase-4, caspase-5 and caspase-11 is an essential step for pyroptosis in activated innate immune cells and endothelial cells stimulated by cytosolic LPS (Gao et al. 2018). This study illustrated that LPS activates NLRP3 by inducing caspase-1 dependent inflammation in pyroptosis. AC inhibited caspase-1 and downregulated the expression of GSDMD, demonstrating that LPS induced pyroptosis via the NLRP3 inflammasome/caspase-1 pathway.

This study has some limitations. First, there are limited replenishment experiments, so how AS-IV upregulates Bcl2/BAX expression ratio could be further explored. Second, there was no animal experiment to prove that AS-IV protects the blood vessels during actual sepsis. Studies are needed to address these limitations.

## Conclusions

**Figure 4.**
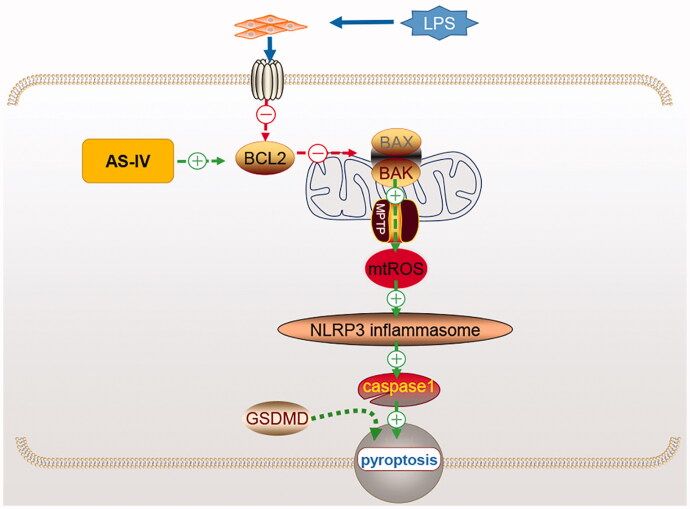
Schematic model of mitochondria and NLRP3 in LPS-induced endothelial injury. The mitochondria might regulate the inflammatory response by upregulating the BCL2/BAX pathway and decreasing pyroptosis in HUVECs via the NLRP3 inflammasome.

This study showed that the NLRP3 inflammasome is highly expressed in HUVECs induced by LPS. The mitochondria modulate ROS production and inflammation response by activating the BCL2/BAX signalling pathway (Figure 4). ROS/NLRP3-mediated inhibition of the inflammatory response induced by AS-IV can significantly suppress pyroptosis in LPS-activated HUVECs. The results of this study imply that AS-IV may be a potential candidate for the treatment of LPS-induced endothelial injury, while the mitochondria and NLRP3 inflammasome might serve as potential therapeutic targets.

## Data Availability

The data supporting this study’s findings are available from the corresponding author LH Guo, upon reasonable request.

## Geolocation information

Foshan, Guangdong Province, China: longitude: 113.12; latitude: 23.02; Guangzhou, Guangdong Province, China: longitude: 113.27; latitude: 23.13.
